# The Importance of Relationships in Patients with Irritable Bowel Syndrome: A Review

**DOI:** 10.1155/2012/157340

**Published:** 2011-12-01

**Authors:** Mary-Joan Gerson, Charles D. Gerson

**Affiliations:** ^1^New York University Postdoctoral Program in Psychotherapy and Psychoanalysis, 80 Central Park West, Suite C, New York, NY 10023, USA; ^2^Division of Gastroenterology, Mount Sinai School of Medicine, 80 Central Park West, Suite B, New York, NY 10023, USA

## Abstract

Chronic illnesses such as irritable bowel syndrome are not experienced by patients in isolation. They live in a context of relationships, including spouses and partners, other family members, friends and business associates. Those relationships can have an effect, both positive and negative, on the course of illness and may also be affected by the experience of living with a chronic illness like IBS. We review the general literature regarding the effect of relationship factors on chronic illness followed by a focus on IBS symptomatology. We then discuss the challenges experienced by partners of IBS patients, followed by the effects of spousal violence, the particular relationship of mothers with IBS and their children, the effects of social support, and the importance of family dynamics and IBS. The final segment includes conclusions and recommendations. The topic, relationships and IBS, may have a significant effect on the lives of IBS patients and deserves more attention than it has received.

## 1. Introduction

Irritable bowel syndrome (IBS) is recognized as an illness in which symptoms may be caused by biological, psychological, and social factors. This paper will focus on the effect of a patient's relationships (family, friends, and work) on the IBS illness experience. This is a subject that has not received sufficient attention in the medical literature. IBS does not occur in a vacuum but in a context of interactions with others. Being aware of those interactions, by the patient and by the physician, may improve patient care.

## 2. Relationships and Health

There is an extensive literature regarding the effect of intimate relationships on health status. An excellent review of marriage and health by Kiecolt-Glaser and Newton [1] describes a number of conditions such as rheumatoid arthritis and hypertension as well as immunological function that may be affected directly by a marital relationship. Of course, this effect may be both positive and negative. Hostile communication in marriage appears to have a deleterious effect on health, in contrast to positive or problem-solving behavior [2]. Effects are more significant for women than for men. For example, one study explored the effect of marital discord on mortality and found that equality in decision making and companionship in marriage were protective against mortality in women, while there was no such effect in men [3]. Why might women be more vulnerable to the effects of conflict in marriage than men? Women are more focused on the relational aspects of marriage than men and are more attuned to its emotional quality. Thus, they are more greatly affected by hostility and problems within a marriage [4]. Being unhappy in marriage may result in psychological problems such as depression, which may adversely affect health status [5]. This effect appears to be “bidirectional” with poor marriages increasing depression and depression decreasing the quality of the marriage [6]. 

Pain is one of the dominant symptoms of IBS. Studies of patients with low-back pain suggest that the relationship between pain solicitousness on the part of a spouse may have beneficial effects in a happy marriage but adverse effects in an unhappy marriage [7]. Open communication is extremely important in relation to pain management. A coping skills training program for couples focused on women suffering from arthritic knee pain which emphasized communication about the illness experience, resulted in less physical disability and pain behavior in the wife after one year, compared to a control group [8].

### 2.1. The IBS Patient and Family Relationships

The association between the quality of intimate relationships and the severity of IBS symptoms has been studied utilizing the Quality of Relationship Inventory (QRI), a validated 25-question self-report measure [9]. The QRI has three stems, support, depth, and conflict. It was included in an international survey that addressed psychosocial cross-cultural issues in IBS [10]. The eight-country survey included New York City, Mexico City, Montreal, England, Bari, Italy, Beersheva, Israel, Kolkata, India, and Beijing, China. 239 patients completed four questionnaires including the QRI, a Mind-Body Attribution Scale, an SF-36 quality of life scale, and the Bowel Symptom Scale (BSS). The BSS Likert scale included self-report of the major symptoms of IBS, abdominal pain, diarrhea, constipation, and bloating. In one report, patient and physician SSS scores were compared and there was no significant difference [11]. QRI results for support, depth, and conflict were correlated with BSS symptom severity. The hypothesis was that support and depth would be positive attributes, while conflict would have a negative impact on IBS.

The results confirmed the hypothesis. Aggregate results for all eight study sites showed that relationship support and depth correlated with lower symptom scores (*P* < .01). In contrast, relationship conflict correlated with higher symptom scores (*P* < .001). The support and depth results can be interpreted in two ways. Clearly, support and depth may be beneficial to the patient and lead to lower symptom scores. However, a patient with lower scores may elicit less anxiety or distress from intimate others, which could access greater support and depth of connection. Through multiple regression analysis, it was found that conflict was a unique predictor of higher scores (*P* = .01). This result is consistent with the marital studies mentioned above but this was the first publication to show an effect of relationship quality on symptom severity in IBS.

The strength of this study included the relatively large number of subjects and the strength of the statistically significant differences. A possible weakness was the inclusion of IBS patients from many different geographic locations where untested variables may have varied considerably, affecting relationship quality in different ways. Also, most patients were seen at tertiary centers, so the results may not apply to nonconsulters with milder forms of IBS.

The Quality of Relationship Inventory has been used in one other IBS study, in an attempt to see whether relationship issues might affect response to treatment. 75 IBS patients entered a biweekly seven session group of IBS hypnotherapy treatment program and completed the QRI before initiation of therapy [12]. Treatment consisted of a gut-focused hypnotherapy protocol developed by Whorwell et al. [13] and modified by Palsson [14]. The QRI was administered to see whether support, depth, or conflicted intimate relationships might impact the responsiveness of a patient to hypnotherapy in a group setting. The hypothesis was that relationship support and depth might enhance responsiveness to hypnotherapy in a group setting, while conflict might have a negative impact. The patients were followed for one year after the final therapy session. They completed the IBS Symptom Severity Scale (SSS) [15] before treatment began and twelve months later. The SSS measures improvement or worsening of symptoms at the one-year mark was correlated with the QRI results. Contrary to the hypothesis, relationship conflict correlated significantly with improvement in symptom score (*P* = .07). 

While this might seem counterintuitive, it is possible that patients experiencing conflict in their personal relationships may represent a group of patients who can benefit from the calming effect of hypnotherapy, reducing their sensitivity to conflict. In addition, they might have found the supportive environment of the group process to be beneficial, making them more responsive to hypnotherapeutic ideation.

Strengths of this study included a relatively large number of subjects, long-term followup, and a high return rate (>90%) of questionnaires at the one-year mark. Weaknesses included lack of a control group without hypnotherapy and the fact that relationship quality was a secondary goal of the study.

### 2.2. Partners and IBS

The focus of another research study was the degree of burden experienced by the partner of an IBS patient. Wong et al. [16] administered the Zarit Burden Interview as well as the Relationship Satisfaction Scale to 152 partners (mostly male) of IBS patients. A healthy control partner population was included in this study. The goal was to determine the degree of experienced burden, how burden was affected by severity of the patient's symptoms, and whether relationship satisfaction and sexual satisfaction were also affected by having a partner with IBS. Results showed that burden scores for IBS partners were double the score of a control partner population (*P* = .0002) and were significantly correlated with disease severity in the IBS partner. Partner satisfaction and sexual satisfaction also correlated significantly with burden scores, but this was found in the control population as well, so this is not unique to IBS.

In one other report, 3090 subscribers to an IBS publication, 86% of whom were diagnosed with IBS by their physician, were mailed a questionnaire that included questions about personal relationships [17]. Of the 1595 subjects who responded, 75% were either married or cohabiting. Of that population, 6% felt that having IBS affected their partner's love and consideration for them, 19% felt that their partners had difficulties with their physical relationship, and 45% thought that IBS interfered with their sex life. These results, though representing a large sample, must be read with caution because there was only a 50% response rate and IBS diagnosis was based on self-report without verification. 

### 2.3. The IBS Patient and Spousal Violence

A unique population study in Nicaragua examined the effect of spousal violence (sexual and physical), on the prevalence of IBS [18]. A number of studies have demonstrated that a childhood history of sexual abuse is more common in patients with IBS than in controls [19]. It should be noted that this is not unique to IBS since the same association has been reported in patients with inflammatory bowel disease [20]. In the Nicaraguan study, a randomly selected representative sample of 960 women were surveyed utilizing questionnaires that documented Rome II criteria for the diagnosis of IBS, as well as a measure of intimate partner violence. Physical violence was found in 14.8% and sexual violence in 4.4% of responders. Comparing women with and without IBS, there was a significant difference in violence history with odds ratios of 2.08 for physical and 2.85 for sexual abuse. 

Strengths of this study included a large number of subjects, the use of personal interviews, and a random sampling technique. Since Nicaragua has had a particularly traumatic history, with prolonged armed struggle against both the dictator, Somoza, and against the Contras, these results may not be applicable to other countries in the developing world. 

### 2.4. Mothers with IBS and Their Children

The effect of maternal IBS illness on children has been studied. Beyond the influence of familial genetics [21], the development of IBS in children and other illness behavior is seemingly influenced by the relationship between a mother with IBS and her children. In an important study [22], 208 mothers with IBS and their 296 children (average age 11.9 years) were compared to 241 mothers without IBS and their 335 children. Children and mothers were independently interviewed and assessed for stress experience, psychological status, level of pain, and coping skill. Mothers also completed a measure of solicitousness. Questionnaires to children were administered orally. When children with mothers with IBS were compared to children with healthy mothers, there was increased frequency of stomach aches, non-GI physical complaints, more time lost from school and more frequent physician visits in the IBS group. Maternal solicitousness was not associated with the presence or frequency of stomach aches but it was correlated with school absence. Solicitousness also did not effect non-GI illness reporting. 

It is psychologically understandable and predictable that mothers suffering from IBS would be more sensitive to, and aware of, gastrointestinal discomfort in their children, but this sensitivity might lead to an increased focus on gastrointestinal complaints voiced by their children. Moreover children of mothers with IBS may feel identified with their mother's discomfort or may inevitably absorb anxiety due to IBS distress. Thus far, there have been no reports on fathers with IBS and their children.

### 2.5. Social Support and IBS

There have been other investigations of IBS and relationships, mostly limited to the realm of general social support, in contrast to intimate relationships. Social support describes the network of resources available to someone when they are facing difficulties in life and expands beyond intimate relationships to friends, neighbors, colleagues, and community. 

In one publication regarding social support and IBS, Jones et al. [23] asked patients to complete a composite measure of social support, the Interpersonal Support Inventory List. This is a 40-item measure that has been validated in other health research studies. The patient population consisted of 74 patients with IBS (out of 145 recruited), 48 patients with inflammatory bowel disease (out of 74 recruited), and 55 controls. Both the IBS group and the IBD group had significantly lower scores than the controls, indicating poor social support. It is noteworthy that there was no significant difference between IBS and IBD patients. There was also no significant difference in psychological disturbance or quality of life suggesting that IBS and IBD have similar psychosocial profiles. It should be noted that the results were cross-sectional, not sequential, so it is not known whether having IBS affects social support or vice versa. 

Another study compared social support in IBS and chronic headache [24]. A structured interview technique was employed and patients were asked about helpful and unhelpful support from intimate others, family members, friends, acquaintances, and physicians. Support was divided into tangible assistance such as information and practical advice and emotional support. Both groups of patients found tangible support to be more helpful than emotional support. However, tangible assistance was less important to IBS patients than those with headaches, which the authors suggest may be related to the embarrassing nature of IBS symptoms. 

The finding that social support is reduced in chronic illness may represent the acute needs that a patient experiences, which may exceed ordinary social relationships. On the other hand, reduction in social support might indicate the difficulties that healthy people experience in relating to someone with a chronic illness, such as anxious identification with illness, a sense of guilt about being well, or perhaps unresolved childhood difficulties in dealing with parental illness. 

### 2.6. Family Dynamics and IBS

The first study that incorporated an investigation of family relationships affecting symptomatology in IBS was a collaborative psychologist-gastroenterologist treatment program, using a biweekly three session model based on discussion between patient and both professionals [25]. Sixteen patients with IBS entered the therapy are of the study and were compared to 8 IBS controls, who received usual medical treatment. During the course of the three-session therapy, it became clear that a patient's relationships had an effect on their illness experience. Examples included spousal conflict, poor personal health care on the part of a parent during childhood, and the occasional eliciting of a history of abuse during childhood. For example, one patient reported highly dismissive attitudes on the part of his spouse who discredited the physiological reality of gut hypersensitivity. Another patient described the pervasive anxiety of her childhood in which a diabetic parent suffered multiple medical crises, which, it seemed, left her with a high level of anxiety about managing medical illness. The novel use of a three-generational genogram during one of the sessions was very revealing, exposing not only psychological dynamics, but also the frequency of chronic gastrointestinal symptoms in other family members. An important part of the treatment was an exploratory discussion of relationship issues, which appeared to contribute, at least in part, to the success of this program. 

The collaborative model proved to be highly successful, resulting in a statistically significant decrease in IBS symptoms, as measured by a two-week daily diary [26], comparing results before onset of therapy to three months after termination. All three major symptoms of IBS, abdominal pain, diarrhea, and constipation were reduced (*P* < .05), as well as global improvement, measured by self-assessment (*P* < .0002). Results were also significantly better than the medically treated IBS control group (*P* < .05). 

Strengths of this study included a medically treated control group, the use of a two-week daily diary for recording of symptoms as well as a global assessment scale, and, a three-month followup. The main weakness was the small number of subjects.

One of the conundrums of IBS research concerns the measurement of symptoms. In the above three studies, three different measures were used. All three have been validated with high reliability quotients [11, 15, 26]. While the two-week daily diary is the most rigorous, it is also more burdensome on the patient than the other two. The SSS is more comprehensive than the BSS and is currently the most commonly used symptom measure.

Insights from this collaborative model were then applied to a successful five-session group program conducted by both a clinical psychologist and a gastroenterologist [27]. One session was led by the gastroenterologist, two by the psychologist, one by a nutritionist, and the last session by both psychologist and gastroenterologist. The psychologist emphasized the importance of context, that IBS is a multiperson phenomenon. Some patients clearly benefited from supportive relationships, while others suffered from accusatory or blaming interactions such as “Can't you relax more?”, or “You're eating all the wrong foods!”. One patient talked about a scenario that took place when she and her husband were about to get into their car when he would ritualistically ask whether the patient was ok or whether she wanted to use the bathroom. While the husband thought he was being helpful, he was actually increasing the patient's anxiety regarding the car ride and interfering with her own self-regulation. These examples demonstrate the complexity of relationship effects on IBS. A three-generational genogram was employed again, including observations about how family members coped with illness. 

A pilot study of one of the groups found statistically significant improvement in IBS symptoms at the end of the five sessions (*P* < .05). However, this involved a small number of patients with no long-term followup.

## 3. Conclusions and Recommendations

These reports, while few in number, support the premise that relationships may have an impact on the IBS illness experience and may even have an effect on response to treatment. The findings reviewed require rigorous confirmation and extension into other areas of research. What are the parameters of relationships that affect the psychological status of a patient? Is compliance with treatment affected? Can couple or family counseling improve the clinical course of the IBS patient?

What recommendations should be made on the basis of all of these studies? Doctors should enquire whether family members and intimate others are sufficiently educated about IBS, and whether they are supportive to the patient or whether there are problems that can be addressed. At the least, it may be helpful for IBS patients to understand that people who are close to them can have a positive or a negative effect on their symptoms. In some circumstances, it may be helpful to have family members present during a consultation. Finally, if it is clear that family relationships are detrimental, or if there is partner confusion about how to be more helpful, referral to a family therapist should be recommended. Family treatment is generally time limited and is highly effective in mobilizing coping strengths.
